# Association of Interleukin-4 Receptor α Chain I50V Gene Variant (rs1805010) and Asthma in Iranian Population: A Case-control Study

**DOI:** 10.2174/0118743064266613231123103523

**Published:** 2024-01-10

**Authors:** Masouma Mowahedi, Azam Aramesh, Mozhgan Sorkhi Khouzani, Marjan Sorkhi Khouzani, Saeed Daryanoush, Mohammad Samet, Morteza Samadi

**Affiliations:** 1 Abortion Research Center, Research and Clinical Center for Infertility, Shahid Sadoughi University of Medical Sciences, Yazd, Iran; 2 Thalassemia and Hemophilia Research Center, Shahid Dastgheib Hospital, Shiraz University of Medical Sciences, Shiraz, Iran; 3 Cellular and Molecular Biology - Genetic Center, Falavarjan Azad University, Isfahan, Iran; 4 Shahid Sadoughi University of Medical Sciences, Yazd, Iran; 5 Department of Immunology, School of Medicine, Isfahan University of Medical Sciences, Isfahan, Iran

**Keywords:** Asthma, I50V, IL-17A, IgE, Elisa, Respiratory disorders, Chronic airway inflammation

## Abstract

**Background:**

Asthma is one of the respiratory disorders caused by chronic airway inflammation. IL-4 has been identified as one of the participating interleukins in the severity of asthma.

**Objective:**

A case-control study was conducted to determine the association of rs1805010, a single nucleotide polymorphism in the interleukin 4 receptor α chain, with asthma and immunoglobulin E and IL-17A serum levels in Iranian populations.

**Methods:**

ELISA was used to investigate the relationship between three different varieties of SNP I50V and serum IL-17A levels, as well as total IgE levels. Based on GINA criteria, patients were classified into mild, moderate, and severe groups based on the association between SNP I50V, IL-17A, and total IgE. In order to analyze the data, the student-t-test and the one-way ANOVA were used.

**Results:**

The SNP I50V was associated with asthma in a significant way (p = 0.001). IL-17A and total IgE levels were significantly higher in asthmatic patients than in control participants (p 0.05 and p 0.021, respectively), but neither showed any association with SNP I50V in the asthmatic patients.

**Conclusion:**

Asthma patients have a higher prevalence of the I allele, reflecting the significance of Th2 cells. Although total IgE and IL-17A levels increased in both disease subgroups, total IgE level augmentation correlates directly with disease severity, while IL-17A level enhancement does not.

## 1 INTRODUCTION

Asthma is one of the most common chronic respiratory diseases and the prevalence of this disease has increased during the last decades [1]. The prevalence of asthma symptoms in Iran is higher than that estimated in the international reports. A systematic review and meta-analysis from Iran show that the highest incidence was 35.4% in Tehran and the lowest was 2.7% in Kerman. The overall prevalence of asthma symptoms at a national level was estimated at 13.14% [2]. It is well established that asthma is a complex disease, and both genetic and environmental factors are responsible for the beginning and progress of this disease. Many studies investigating associations between genetic variants and asthma risk have been published [3]. Different genes predisposing to asthma have been studied within the past years. Studies emphasize single nucleotide polymorphisms (SNPs) in developing susceptibility to the disease [4-6]. It has been proven that there is a relationship between gene regions in the short arm of chromosome 16 (*16p12*) and susceptibility to allergic diseases. One of the products of this gene region is the alpha chain of the interleukin-4 receptor (IL-4Rα) [7, 8]. One of several polymorphisms identified in this gene is SNP I50VIsoleucine-50-valine (I50V) is located at position adenine +4679-to-guanine (A+4679G) [8]. Although investigations have been conducted on this single nucleotide polymorphism, there are still many contradictions, as shown in Table **1**. IL-4 is the major cytokine of the Th2 cells. Th2 cells still play a major role in asthma [7], thus, Th2 cells have been considered a major factor in asthma for many years; this means that Th2 cells could initiate the inflammation in asthma by mobilizing and utilizing other effector cells, including eosinophils and mast cells [9]. Accordingly, asthma was classified into two distinct subgroups:Th2-high and Th2-low. With the introduction of the new subtype of T helper cells (Th17 cells), the key role of these cells was detected in initiating inflammation in some inflammatory diseases. Consequently, the possible role of these cells in stimulating inflammation in asthma was investigated, and neutrophilic inflammation in asthma was attributed to the Th17 cells [10].

Some evidence has been reported in controlling the roles of IL-4 on the Th17 cells and, consequently secretion of IL-17 [17]. Overall, given the participation of Th17 cells and its main cytokine (IL-17) in the pathogenesis of asthma [18, 19], the gene of the alpha chain of the IL-4 was selected in this study. In addition, there is no study on I50V SNP in the Iranian population. Studies on asthma in other populations have been conducted on its effect in terms of allergy without assessing the IL-17. Therefore, in the present study, we investigated the SNPs in asthma disease and measured the serum concentrations of total IgE and IL-17A.

## 2 MATERIALS AND METHODS

### 2.1 Sampling

In this case-control study, the statistical population consisted of 240 patients with asthma as the case group and 110 non-asthmatic people as the control group during 6 months from September 2013 to February 2014in Shahid Sadoughi Hospital, Yazd province, Iran. Sampling was performed by convenience method. The patients were selected out of the people referred to the asthma clinic to treat their disease under the supervision of a specialist. Asthma was diagnosed in these individuals after taking their medical history, doing medical examinations, and performing lung function tests. These people had mild to severe asthma. The type of asthma was determined by a lung specialist based on GINA criteria. GINA classifies asthma severity as 1) Mild asthma, which can be treated with daily/as-needed low dose ICS-LABA with optional leukotriene receptor antagonist (LTRA), 2) Moderate asthma, which is treated with daily low or medium dose ICS-LABA with optional LTRA, and 3) Severe asthma, which is treated with daily high-dose ICS-LABA with optional tiotropium or biological therapy [20], using spirometry and physical examinations. Using invasive ventilation in all the cases was excluded from the study and was not monitored. The case group did not have any other lung disease except asthma. In the present study, 28 cases out of 240 asthmatic cases were affected by mild, 33 cases by moderate, 39 by severe asthma and the status of 140 cases were not available. The control group was selected out of healthy individuals without symptoms of asthma and other pulmonary disorders. These people without any history of allergy, autoimmune disorders, systemic diseases, and tobacco consumption in themselves or their families were chosen as the control group concerning age and sex matching. The patients and controls were enrolled in the study after explaining the purpose of the study, signing the informed consent form, and completing the questionnaire. People who were not willing were excluded from the study. In the case of children, parental consent was required. Ethical approval for this trial was granted by the Shahid Sadoughi University of Medical Sciences Ethics Committee on 17/1/51057.

### 2.2 Sample Preparation

Each subject was given a 5 ml whole blood sample and divided into 2 tubes. A temperature of -80°C was maintained throughout the storage of serum samples until measuring the concentrations of IL-17A and total IgE. 4 milliliters of the whole blood containing EDTA as anticoagulant was obtained and stored at -80°C until DNA extraction.

### 2.3 DNA Preparation

Genomic DNA was extracted from white blood cells using the salting out method, and supernatants were discarded after centrifuging at 4500 rpm for 15 minutes. 1.5 ml of proteinase K buffer was added to the residual white pellet in the residual supernatant (Tris-Hcl, 4mmol Na2EDTA, 100 mmol NaCl, pH 7.8), then 100 µl of 10% SDS was added. Re-suspended and thoroughly dissolved pellets were mixed in the solution. 20 µl of Proteinase K solution was then added, which was refrigerated (20mg/ml). The tubes were soaked for 90 minutes in water at 55C°. 5.3 M NaCl was added and vortexed for 15 seconds after the tubes were placed on ice for 2-3 minutes. A centrifuge was then used for 20 minutes at 4500 rpm. Cold isopropanol was added to the supernatant in equal volumes. Five to six gentle inversions were performed to precipitate DNA, followed by 5 minutes of centrifugation at 4000 rpm. Once again, tubes were centrifuged after \the supernatant was discarded, and ethanol (70%) was added .Re-suspended DNA was mixed with 200-300 µL of distilled water after discarding the supernatant [21]. After extracting the DNA, it was dissolved in water and stored at –80°C until PCR was carried out.

### 2.4 IL-17A and Total IgE Assay

The contents of IL-17A and two commercial ELISA tests were used to determine IgE levels (MABTECH AB, Sweden, Cat No. 3520-1H-6 and Monobind Inc. USA, Cat No. 2525-300 respectively) Following the instructions of the manufacturer. Using an Awareness 2400 microplate reader, the optical density was measured. There was a sensitivity of 0.01 pg /ml for the IL-17A ELISA and 0.125 IU/ml for the total IgE ELISA.

### 2.5 Genotyping

I50V SNP reference is rs1805010 from the National Center for Biotechnology Information. The I50V polymorphism was genotyped by PCR-RFLP analysis using the primer pair 5ʹ-GGCAGGTGTGAGGAGCATCC-3ʹ, 252-233 bp upstream of the Ile50Val polymorphic site and 5ʹ-GCCTCCGTTG TTCTCAGGTA-3ʹ, 399-418 bp, as described previously [22]. The PCR reaction was performed using a programmable thermal cycler (Convergent, Germany, TC96G) after an initial 5 min denaturation at 94°C followed by 35 cycles of 94°C for 30s, 64.7°C for 30s, 72°C for 30s, and final 5 min extension at 72°C. PCR products and RsaI as restriction enzymes were incubated overnight at 37°C. PCR fragments digested with RsaI were electrophoresed in 2% agarose and visualized by ethidium bromide staining and ultraviolet transillumination using Gel Documentation System (Syngen, UK, UGENIUS).

## 2.6 Data Analysis

After collecting the data, all assessments were performed using SPSS 19. The distribution of alleles and genotypes of the studied single nucleotide polymorphisms in both patients and control groups was analyzed using the Chi-square test. The amount of Total IgE in serum and IL-17A in the patient and control groups was assessed by independent t-test, and the ANOVA test was used to evaluate the serum concentrations of Total IgE and IL-17A among the subtypes of asthma. Furthermore, analysis of variance was used to examine the association between single nucleotide polymorphism and serum concentrations of Total IgE and IL-17A. The significance level was considered p<0.05 in all tests.

## 3 RESULTS

The study included 240 patients with asthma and 110 control subjects. Some characteristics of the case and control groups are summarized in Table **2**. In the present study, the geno type of single nucleotide polymorphisms could be determined using the PCR-RFLP technique and based on the number and size of the established bands on gels. Allele A and allele G could be recognized by observing, respectively a band with a size of 273 bp and two bands with sizes of 254 bp and 11 bp (Fig. **1A** and **1B**).

### 3.1 The Results of Genotyping and Single Nucleotide Polymorphisms Alleles of the Il-4Rα Gene

In the present study, single nucleotide polymorphism of I50V in the IL-4Rα gene was investigated (P=0.001). The frequency of alleles and genotypes of I50V in two groups of patients and controls is compared in Table **3**.

In order to investigate the association between these SNPs with asthma severity, genotyping of single nucleotide polymorphisms of I50V in the subtypes of asthma was determined based on the severity of the disease (Table **4**).

### 3.2 Results of Assessing Serum Concentration of Total IgE

**Fig. (2):**
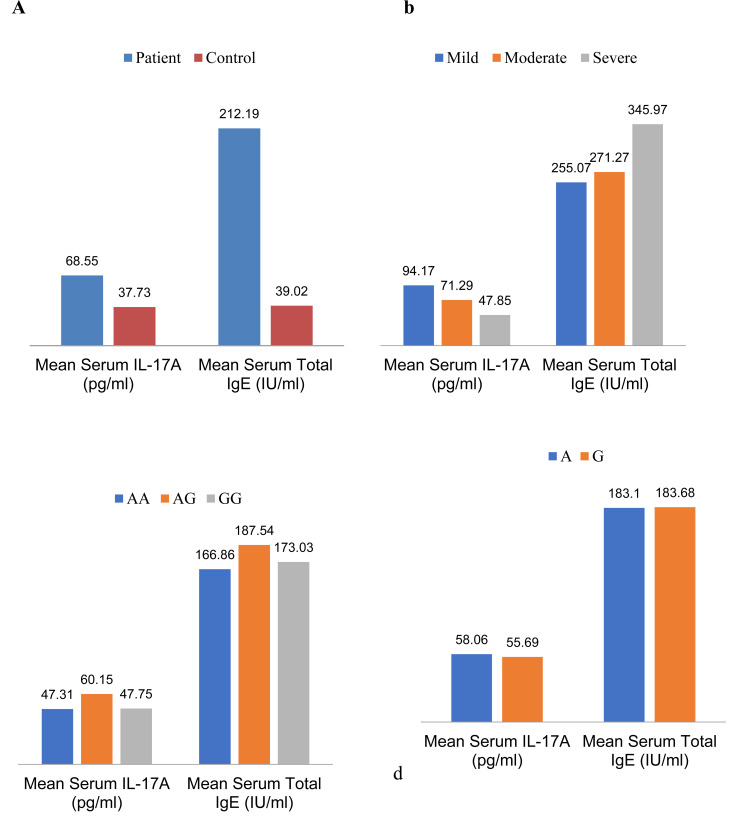
Mean serum IL-17A and total IgE in asthmatic patients and control group (**a**) and subtypes of asthma (mild, moderate and severe) (**b**) and genotypes and alleles of SNP I50V (**c**& **d**).

In this study, serum concentrations of total IgE in patients and controls were evaluated (p <00.1). There was no significant relationship in terms of the severity of the disease in the subtypes of asthma. Moreover, there was no correlation between the SNP of I50V in the IL-4Rα gene and the concentration of total IgE in the serum (Fig. **2**).

### 3.3 Results of Assessing Serum Concentrations of IL-17A

In the current investigation, serum concentrations of IL-17A were assessed in patients and control groups (P=0.026). There was no significant association in terms of disease severity between the subtypes of asthma and the respective polymorphism(P<00.1). In addition, no significant relationship was found between single nucleotide polymorphisms of I50V in the IL-4Rα gene and the amount of IL-17A in serum (Fig. **2**).

## 4 DISCUSSION

Within the late 90s, many studies have been conducted on the SNP I50V. Initially, studies were conducted to evaluate the effect of SNPs on allergic diseases, especially asthma. Some of these researches are summarized in Table **1**.

SNP I50V is located in the coding region ofIL-4R α. As a result, SNP I50V can influence the signal transduction pathway of the receptor, and in fact, it could cause IL-4 receptor dysfunction. The result of this SNP occurrence is fewer responses of the cell to IL-4 [23]. Theoretically, it can be concluded that the I50Vallele has a potential role in susceptibility to asthma and allergic diseases by stimulating the clonal expansion of Th2 cells. However, there is no consensus on the impact of this SNP on allergic diseases and asthma (Table **1**). A decade after identifying the SNP, the type of investigations has changed. The role of this SNP, in addition to asthma and allergic diseases, was also evaluated in other inflammatory diseases, especially in rheumatoid arthritis.

The role of this SNP in rheumatoid arthritis and the possibility of the impact on subtypes of Th2 and Th17 cells were investigated through the identification of Th17 cells [24]. According to research conducted on this SNP in rheumatoid arthritis, it can be concluded that dysfunction of IL-4 receptors could cause impairment on the regulation of the inflammatory Th17 cells through IL-4, which in turn could lead to further differentiation of Th17 cells and disrupt the differentiation of Th2 cell. Consequently, the release of inflammatory Th17 cell cytokines, including IL-17 is increased. As a result, SNP I50V has a negative action, and the I50allele has a protective and anti-inflammatory role in rheumatoid arthritis [25, 26].

Due to the differences like asthma and allergic diseases with rheumatoid arthritis, the role of SNP I50V could be different in these diseases. In asthma and allergic diseases, the SNP I50V can act positively because the I50allele stimulates the development of Th2 cells (patients with asthma were significantly more likely to have the I50 allele than controls, according to the results of this study) (Table **3**). Thus, It appears that the I50 allele may be beneficial for asthma and allergic diseases, as it prevents this stimulation from occurring. In the case of asthma and allergic diseases, the issue is not as simple as rheumatoid arthritis. It can be stated that the Th2 cells have a protective role, and the Th17 cells have an inflammatory role in rheumatoid arthritis. In contrast, both of these two cell lines have inflammatory roles in asthma and allergic diseases.

The evidence of the involvement of Th17 cells (identified as inflammatory cells) in the pathogenesis of asthma and allergic diseases is on the rise [26]. According to the results obtained in this study, despite a higher frequency of the I50allele in asthmatic patients compared to control subjects, IL-17A serum levels are significantly increased along with total IgE in asthmatic patients, suggesting that Th17 cells, although less numerous than Th2 cells, can contribute to asthma pathogenesis (Fig. **2a**).

Previous studies have emphasized the participation of Th17 cells and their cytokines, especially IL-17, in the pathogenesis of asthma. Another important issue inferred from these studies is the possibility of controlling the role of IL-4 on Th17 cells and the secretion of IL-17[27[REMOVED HYPERLINK FIELD]]. Perhaps this controlling role in Th2 cells and the secretion of IL-4 can be generalized for IL-17 as well. Because according to our results, despite the increase in the mean serum level of the Total IgE and IL-17A in the patients with asthma, increased serum levels of Total IgE in subtypes of asthma are associated with decreased serum levels of IL-17A and *vice versa* (Fig. **2b**).

We conducted the present study according to the research performed on rheumatoid arthritis about SNP I50V and considering the negative role of SNP I50V in inflammatory disease of rheumatoid arthritis and the fact that Th2 cells, unlike the protective role in the inflammation of rheumatoid arthritis, are still the main inflammation trigger cells in asthma. The SNP I50V can reciprocally act against asthma. On the one hand, it can trigger the inflammatory process of asthma by stimulating the differentiation of Th2 cells; on the other hand, it can reach the clinical presentation of asthma in another way by stimulating the differentiation of Th17 cells. The results of the present study could verify this claim.

In other words, Compared to the control group, asthmatic patients had a significant increase in total IgE and IL-17A serum levels, which indicated a two-way interaction between Th17 and Th2 cells. They thus suggested a two-way role for SNP I50V in asthma pathogenesis. In asthmatic patients, there was an increase in total IgE and IL-17A serum levels. As found in this study, serum concentrations of total IgE and IL-17A in patients were significantly increased compared to the control patients (Fig. **2**). However, no significant correlation was observed between serum concentrations of total IgE and IL-17A by genotypes and alleles of this single nucleotide polymorphism (Fig. **2-c** and **2-d**). Perhaps, unlike the disease of rheumatoid arthritis whose protective role has been reported in the I50 allele, that is why the results are contrary in asthma.

### 4.1 Investigation of the Correlation between total IgE Levels in Serum and IL-17A with Asthma

Another point that should be considered about asthma is the balance of the subtypes of T cells in the pathogenesis of the disease. It is believed that the Th1 and Th2 cells in a natural status are balanced and the Th17 and Treg cells are so as well. This balance is disturbed in asthma; asthma occurs when Th2 or Th17 cells become dominant [28, 29].

Based on the results of this study, the serum concentration of total IgE was elevated, consistent with increasing disease severity. The mean serum concentration of total IgE in patients with severe asthma had the highest amount, and in patients with mild asthma, it had the lowest rate. In contrast, the serum concentration of IL-17A had a descending line by increasing serum levels of total IgE (Fig. **2**). Since the total IgE is the result of Th2 cell activity and the main cellular source of IL-17A is Th17 cells, and according to our results in this study on the serum levels of total IgE and IL-17A, it can be proposed that the Th2 and Th17 cells are in balance with each other. Clinical symptoms of Th2-related asthma will occur when the balance is toward the Th2 cell^’^s dominance; clinical symptoms of Th17-related will be observed, while this balance is toward the Th17 cell^’^s dominance. However, complementary molecular and cellular techniques should be performed to confirm this proposal, if possible, to isolate the IL-17A secretion of Th17 cells from other sources. There is still much uncertainty about asthma disease. New discovery could open a new window in the treatment process of asthma disease in the future.

## CONCLUSION

The results of this study confirmed that there was a relationship between I50V SNP and asthma. In contrast, no association was found between the genotypes of this SNP and serum concentrations of total IgE and IL-17A in the Iranian population. Another interesting result that we achieved in this study was the inverse association of increased serum levels of total IgE and IL-17A in patients with mild, moderate, and severe asthma. This means that serum levels of total IgE were increased by increasing the severity of asthma, while the serum levels of IL-17A were decreased. More research in this matter seems essential.

## Figures and Tables

**Fig. (1A):**
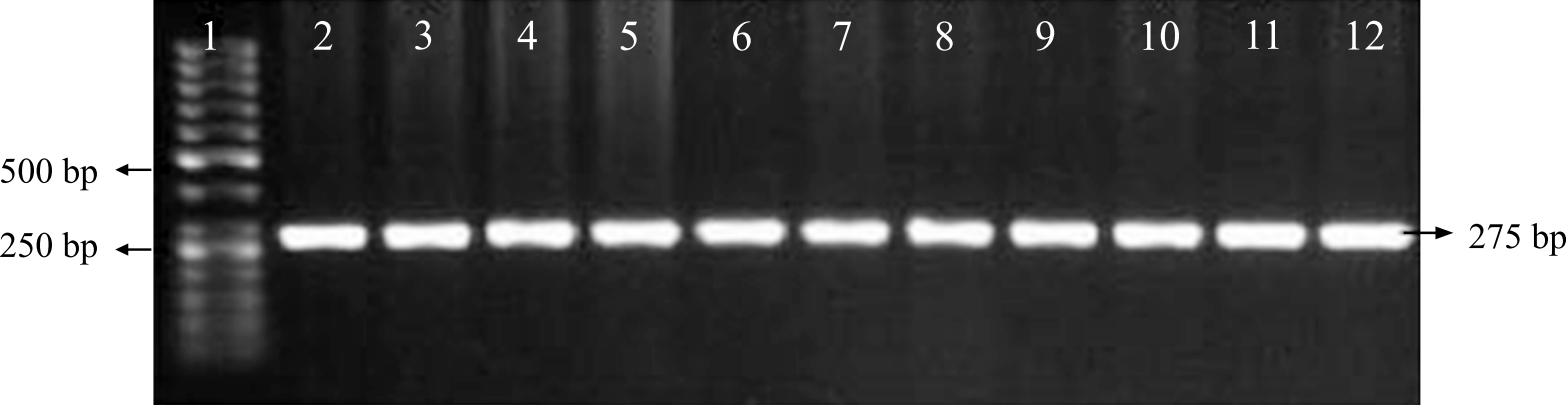
Results of PCR on some samples. PCR products were analyzed by 1% agarose gel electrophoresis, which are shown as single bands with 275 bp and without non-specific bands. The first well is related to 50-bp marker, and the second to twelfth wells are associated with PCR products of the first to eleventh samples.

**Fig. (1B):**
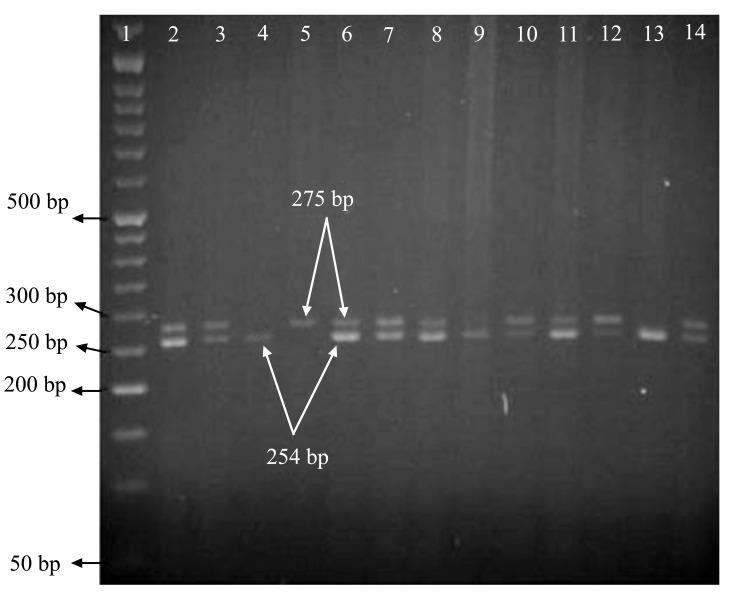
Fragments resulting from enzymatic digestion of PCR. After enzymatic digestion of amplified fragments containing respective single nucleotide polymorphism using RsaI restriction enzyme, if there is allele A, allele G or heterozygote, a fragment with 273 bp, a fragment with 254 bp, and two fragments with 254 bp and 273 bp would be observed, respectively. The fragments resulting from enzymatic digestion of PCR products of I50V single nucleotide polymorphisms after 2% agarose gel electrophoresis are shown in the Figure. The first well contains 50-bp marker, and the fourth, ninth and thirteenth wells have samples with allele G. The fifth well has a sample with allele A, and the heterozygous type is seen in other wells.

**Table 1:** Characteristics of the case-control studies based on studying I50V SNP.

**Result**	**Disease**	**Number of samples (cases/control)**	**Age group**	**Country**	**Year**
Associated with asthma, atopy and IgE synthesis	Atopic asthma	10/10	children and adult	Japan	1999 [11]
Association with asthma and atopy	asthma and atopy	302/392	children and adult	USA	2000 [12]
Associated with asthma and atopy	Asthma and atopy	252/227	children	Chinese	2010 [13]
associated with pediatric asthma but not associated with IgE synthesis	pediatric asthma	240/140	children	Hong Kong, China	2006 [14]
associated with asthma severity	asthma	88/202	children and adult	Brazil	2008 [15]
not associated with allergic rhinitis	allergic rhinitis	54/45	adult	Malaysia	2012 [16]

**Table 2:** Clinical characteristics of asthmatic patients and control subjects.

**p-value**	**Control subjects (n=110)**	**Asthmatic patients (n=240)**	**Parameters**
0.254	53/57 (48.2%/51.8%)	100/140 (41.7%/58.3%)	Male/female sex
0.137	39.96 ± 14.15	42.61 ± 15.96	Age (y) (Mean ± SD)
< 0.01	39.02 ± 41.54	212.19 ± 301.7	Total IgE (IU/mL) (Mean ± SD)

**Table 3:** Frequency of IL-4 receptor α alleles in asthmatic patients and the control subjects.

**P-value**	**Genotypes**			**Alleles**		
**GG**	**AG**	**AA**	**G**	**A**
**0.001**	**49** **(20.4%)**	**149** **(62.1%)**	**42** **(17.5%)**	**247** **(51.5%)**	**233** **(48.5%)**	**Patients** **n=240**
	**42** **(38.2%)**	**48** **(43.6%)**	**20** **(18.2%)**	**132** **(60%)**	**88** **(40%)**	**Controls** **n=110**

**Table 4:** Frequency of IL-4 receptor α alleles in subtypes of asthma.

**Genotypes**	**Asthmatic patients**			
**Mild** **n=28**	**Moderate** **n=33**	**Severe** **n=39**	**NA٭** **n=140**
**AA**	**2** **(7.1%)**	**1** **(3.0%)**	**2** **(5.1%)**	**37** **(26.4%)**
**AG**	**19** **(67.9%)**	**24** **(72.7%)**	**27** **(69.2%)**	**79** **(56.4%)**
**GG**	**7** **(25.0%)**	**8** **(24.3%)**	**10** **(25.7%)**	**24** **(17.2%)**
**P-value**	**0.965**			-

٭NA: not available.

## Data Availability

The data and supportive information are available within the article.

## LIST OF ABBREVIATIONS

SNPs: Single Nucleotide Polymorphisms
IL-4Rα: Interleukin-4 Receptor
LTRA: Leukotriene Receptor Antagonist
